# How the term “white privilege” affects participation, polarization, and content in online communication

**DOI:** 10.1371/journal.pone.0267048

**Published:** 2022-05-04

**Authors:** Christopher L. Quarles, Lia Bozarth

**Affiliations:** School of Information, University of Michigan, Ann Arbor, Michigan, United States of America; St John’s University, UNITED KINGDOM

## Abstract

The language used in online discussions affects who participates in them and how they respond, which can influence perceptions of public opinion. This study examines how the term *white privilege* affects these dimensions of online communication. In two lab experiments, US residents were given a chance to respond to a post asking their opinions about renaming college buildings. Using the term *white privilege* in the question decreased the percentage of whites who supported renaming. In addition, those whites who remained supportive when *white privilege* was mentioned were less likely to create an online post, while opposing whites and non-whites showed no significant difference. The term also led to more low-quality posts among both whites and non-whites. The relationship between question language and the way participants framed their responses was mediated by their support or opposition for renaming buildings. This suggests that the effects of the term *white privilege* on the content of people’s responses is primarily affective. Overall, mention of *white privilege* seems to create internet discussions that are less constructive, more polarized, and less supportive of racially progressive policies. The findings have the potential to support meaningful online conversation and reduce online polarization.

## Introduction

Billions of people use the internet and social media as a window to the world. Rather than being made of glass, this window is manufactured and shaped by the collective choices and language of billions of people. Online behavior is shaped by a community’s language [1], norms [2], moderation policies [3], initial posts [4], and the perceived demographic and social status of the participants [5].

This study aims to understand how the content that is posted online is affected by one particular piece of controversial language: the term *white privilege*. While the term *white privilege* existed in academic writings as early as the 1980s [6], the general public has become increasingly aware of it amid the heightened racial tension of the past decade [7]. At the same time, social media has increased the availability of extreme, and often vitriolic, views online. A search for “white privilege” on any major social media platform will show a range of posts representing strong feelings from multiple ideological angles.

Social media has given people more options than ever for how to spend their time. Individuals today can scroll through a near-infinite stream of cat videos or talk about their favorite video game instead of engaging in uncomfortable discussions of race. Small changes in initial language have the potential to create large effects in both the content that gets posted and the traits of those engaged. To understand the effects of the term *white privilege* on social media discussions, we ran two experiments in a simulated online environment. Respondents were asked, “Should colleges rename buildings that were named after people who actively supported X?” where X is either *racial inequality* or *white privilege*. We studied *how* people responded by looking at stance (pro/con), the frames (arguments, topics, and ideas) used in the response, and response quality. We also examined *who* would respond to the post by looking at both stated and actual likelihood of response. In addition, we use the posts to simulate the composition of responses in a real online forum.

### How people respond to white privilege

Privilege is “unearned advantage derived from one’s group membership” [8]. In the present study, white privilege refers to racial privilege in the American context. The concept of white privilege is central in areas such as contemporary diversity training [9] and whiteness studies scholarship [10]. However, in public discussion, the term is more controversial. Popular media has variously talked about white privilege as a topic to be taught to children [11], a racist term [12], and a distraction from the root causes of racial inequality [13]. To be clear, this study does not directly examine the concept of white privilege itself, or whether whites think they have advantages due to their race. Instead, our goal here is to look at behavior: How individuals respond to the *term* in the context of an online forum. We expect that whites will respond differently to the term *white privilege* than other groups for two reasons.

Social identity theory suggests that we often define ourselves, and others, in terms of the groups that we are members of [14]. A person’s behavior or perception of their social status might change based on which group membership is most salient at the time [14]. The term *white privilege* evokes images of whites as a coherent group with representative traits. So we expect that the term will lead to increased salience of racial identity among whites, which will affect their responses.

In addition, whites have different views, on average, than members of other races about the advantages that whites have. In a recent Pew study, 47% of whites said that whites benefit either a great deal, or a fair amount, from advantages that Blacks don’t have [15]. In contrast, 89% of Blacks and 74% of Hispanics said that whites benefited from these advantages. While this difference in perception may come from motivated reasoning [16] or from genuinely different life experiences [6], by itself it is likely to affect how whites respond to the term *white privilege*.

Some individuals identify more strongly with their race than others. The strength of this pre-existing identification can give a differential effect on responses to racial priming, which has been shown in a variety of contexts with a variety of identities [17–19]. American whites have repeatedly shown less identification with their race, on average, than other groups [20], likely because being in the minority reinforces category differences and increases the salience of racial identity [21, 22]. However, whites vary in the strength of their racial identity, and this affects their thoughts, feelings, and behavior [23]. While the current study does not include a measure of strength of racial identification, it is reasonable to expect that different groups of whites may respond differently to the term *white privilege*.

Responses to the term *white privilege* do not come purely from a place of reasoned disagreement. One meta-study found that emotions were twice as important as beliefs in predicting discrimination [24]. Just like we can define ourselves using group stereotypes [25], the theory of intergroup emotions describes how group membership can cause us to feel emotions [26]. Anger has been shown to mediate the effects of perceived injustice on retributive action [27]. And guilt has been shown to mediate framing effects on support for Dutch-Indonesian reparations [18] and on perceptions of American racial inequality [28] among members of the dominant group. Those emotions do not stop when people go on social media [29]. Since discussions of white privilege create uncomfortable feelings among some people, these heightened race group-based emotions may cause individuals to avoid engaging in online discussions.

### Online conversations

Online information plays a significant role in shaping twenty-first century society. From the 24-hour clickbait-based news cycle, to discussion forums with infinite scrollers, to group-based conversations with friends on messaging apps, online media affects how we think about current events [30], who our friends are [31], and how we feel about ourselves [32]. However, our perceptions built using the online world don’t always represent reality [33, 34]. The artificial reality we see online is sensitive to affordances and moderation policies of individual platforms [3, 35] and is highly dependent on initial conditions [4]. In addition, media consumers interpret what they read based on pre-existing beliefs and biases [36]. Ultimately, online media enables different groups of people to have very different perceptions of truth. Race is especially problematic in this respect, since differences in offline lived experiences have the potential to create barriers to a shared reality. We look at that online reality by examining four individual-level dimensions: *avoidance*, *conversation quality*, *stance* (support or opposition towards a topic under discussion), and the *frames* that are used in responses. To understand the system-level impressions of public opinion on a real discussion forum, we also examine the overall *composition* of posts.

#### Avoidance

Individuals’ decisions about *whether* to participate in discussions play a central role in the social media landscape. Individuals avoid posting for a variety of reasons, including lack of time or interest, concern about offending someone or giving a bad representation of themselves [37]. Individuals are also less likely to share negative and emotion-laden content [38], and are less likely to post in general if they are female, afraid of isolation, didn’t feel strongly, or felt like their opinion didn’t match the way the country was moving [39]. While avoidance has the potential to be protective of social relationships, it can also lead to adverse personal effects from stifling expression [40]. More systemically, avoidance is a key component of the “spiral of silence” [41], which leads to perceived minority opinions being underrepresented on social media [42]. Of course, the vast majority of social media consumers are lurkers–people who consume content without contributing [43]. And even regular posters read more than they post. In the context of race, people have been shown to distance themselves from sources of identity threat [44]. So we expect that whites will be more likely to avoid responding to the *white privilege* question, particularly those whites who might feel like their ideas are in the minority or who experience identity threat.

#### Conversation quality

Incivility and toxicity are important metrics for online spaces, and race-related topics are more likely to draw uncivil comments [45]. Even if posts can be categorized as civil, they may be confusing or add little to the conversation. So we operationalized a *low-quality* response as one that attacked people, challenged the question itself, contained little content, or was hard to understand. Given the toxic nature of some online conversations around race [46] and the discomfort many whites have with the concept of white privilege [15, 16], we expect that the term will lead to lower average conversation quality among whites.

#### Stance & frames

We measure the content of a post in two ways. **Stance** describes whether an individual supports or opposes the proposed topic. We also look at the topics, or arguments, mentioned in each response. These could be described as the *ideas* that the writers have about the topic. Alternatively, if we think of social media consumption, those same ideas become a way of **framing** the conversation. In this paper, we will use the term *frames* to describe this concept.

In the current context, we know that many whites do not believe they have race-based advantages [15]. The idea of *white privilege* is not consistent with their understanding of the world. Consequently, we hypothesize that fewer whites will be supportive of renaming building when *white privilege* is brought up.

Note that *stance* and *frames* are separate, but highly related. Supporters of a proposition typically find certain frames more salient than opponents do. For instance, abortion opponents often frame the procedure as ending a life, which puts the fetus at the center of attention. While pro-choice advocates tend to frame the issue around the needs and rights of the mother. Speakers and writers will influence support for a topic by framing the issue in different terms [47]. In our experiments, we expect treatment condition to influence both stance and frames. Previous work suggests that that *white privilege* will have a primarily affective effect on individuals [24, 48]. We expect this blunt mechanism to influence stance, instead of the frames used in complex reasoning. In this case, frame use would arise from motivated reasoning, as individuals tried to explain the stance that they had already chosen. So we hypothesize that there will be no significant difference in frames after controlling for stance.

#### Composition of posts

Social media is used by individuals [49], researchers [50], journalists [51] and policy makers [52] to understand public opinion. However, responses on social media are not usually representative of the population as a whole [53]. Online behavior depends on the community members, the affordances of the forum, and framing. To understand how the term *white privilege* affects this perception, we summarize the **composition** of responses in each treatment condition. By this we mean the set of responses, taken as a whole, as a reader might perceive them. Unlike the other four dimensions, which focus on individual behavior, this variable describes the system’s behavior. For instance, does an online community seem supportive of renaming buildings? Or does the community seem to oppose it? This composition can also create higher-order effects on the community, as individuals make decisions about what to post [37, 54]. Given the relatively strong responses to the term *white privilege* online, and the lack of debate about whether racial equality is an important social value in the U.S., we expect that *white privilege* and *racial inequality* will create simulated communities with different compositions.

In summary, the literature suggests the following hypotheses:

**Hypothesis 1 (Avoidance):** Whites will be less likely to respond when asked about *white privilege*.

**Hypothesis 2 (Stance):** Whites will, on average, be less supportive of renaming buildings when asked about *white privilege*.

**Hypothesis 3 (Conversation Quality):** Whites will, on average, have lower quality responses when asked about *white privilege*.

**Hypothesis 4 (Frames):** Supporters and opponents of renaming buildings will bring up different sets of frames. And, after controlling for support, asking about *white privilege* will not affect the frames used.

While not a formal hypothesis, prior work suggests non-whites will either show no mean difference between treatment conditions in these first four dimensions, or show a trend in the opposite direction from whites. Overall, the first four hypotheses should lead to:

**Hypothesis 5 (Composition of responses):** In an online conversation, the use of the terms *racial inequality* and *white privilege* will result in a different composition of posts.

### Study design

We explored these hypotheses through two experiments. Experiment A enabled us to gather responses from both individuals who would have posted online and those who would have self-censored. Because Experiment A asked people to self-rate their likelihood of responding, Experiment B examined revealed preferences by giving respondents a choice of questions to answer. A lab experiment was chosen to isolate the effects of language, avoid higher-order network effects on peoples’ responses, and ensure that we could gather data about people who would otherwise avoid responding.

### Respondents

Participants were US residents, drawn from Amazon Mechanical Turk (MTurk), who had completed 1000 tasks with 98% or higher acceptance rate. Both experiments were listed as the same task in the MTurk system. US resident MTurkers have been shown to be generally representative of the national population [55]. Participants were randomly assigned to experiment (A or B) and to treatment condition (*racial inequality* or *white privilege*). After excluding respondents who did not respond to the prompt, we were left with 478 people in Experiment A and 446 in Experiment B. Descriptive statistics about the sample are in Table 1.

**Table 1:** Demographics of respondents.

	Experiment A		Experiment B	
	Racial Inequality	White Privilege	Racial Inequality	White Privilege
Number of Respondents	250	228	233	213
Male	51%	53%	56%	50%
Female	48%	46%	43%	49%
White	82%	78%	81%	84%
Black	11%	8%	6%	8%
Asian	6%	13%	9%	6%
Hispanic/Latino	6%	6%	5%	5%
Other	2%	2%	3%	3%
Multiracial	7%	7%	6%	7%
Bachelor’s Degree	59%	57%	67%	65%
Politics				
Mean	-0.42	-0.35	-0.37	-0.44
Standard Deviation	1.2	1.2	1.2	1.2

Politics was rated on a scale from -2 = *strongly liberal* to 2 = *strongly conservative*. Race percentages add to more than 100% because some people identified as multiracial.

We expected that people who identified *only* as white (74%) would tend to respond differently to the term *white privilege* than those who identified, at least in part, as a member of another race. To describe this latter group, we use the term *non-white* to signify that we don’t expect them to have the same white identity as those who identify as only white. Four respondents did not provide a race. They are included in any analyses which don’t involve race.

### Instrument

Respondents in both studies received an online survey broken into two parts. After giving informed consent, respondents were sent to the Part 1 that corresponded to their experiment. In Part 1, each respondent was randomly assigned one of the two questions: “Should colleges rename buildings that were named after people who actively supported racial inequality?” or “Should colleges rename buildings that were named after people who actively supported white privilege?” The question language was chosen based on conversations with colleagues and vetting interviews during the study design phase. We purposely tried to use general language that might evoke a broad, identity-based response. *Racial inequality* was chosen as a counterpoint to *white privilege* because it seemed less likely to increase the salience of racial identity. Equality is an American ideal that we thought most respondents would support. And the topic of renaming college buildings seemed to give enough opinion diversity to see meaningful differences in the data.

In Part 1 of Experiment A, each respondent was randomly shown either the *racial inequality* or *white privilege* question. They were then asked: (a) “How likely would you be to respond to this question if you saw it in an online community?” and (b) “If you did reply to this question, what would you post in the online forum? Write the reply exactly as you might post it online.” Responses to (a) were on a 5-point Likert scale from *very likely (2)* to *very unlikely (-2)*. Responses to (b) were free-written into a text box. After submitting Part 1, respondents were sent to Part 2.

Each participant in Experiment B was also randomly assigned to either the *racial inequality* or the *white privilege* condition. However in this case, for Part 1 participants were given the choice of two questions in a randomly chosen order. They were told that they could respond to either question, but only one. The questions were the renaming-buildings question (which depended on their treatment condition): “Should colleges rename buildings that were named after people who actively supported *racial inequality/white privilege*?” and the college-loans question: “Should college tuition loans be forgiven for people who choose to go into public service, such as social workers and teachers?” The college-loans question was chosen to avoid race and provoke a similarly diverse range of opinions. Text responses to the college-loans questions were not coded or used. After responding to their chosen question in a text box, respondents were sent to the same Part 2 as in Experiment A.

The benefit of the design of Experiment B is that it elicits behavior in a way that better approximates a real social media site. Attention is a precious commodity online. Ads and posts vie for time on consumers’ screens. The option of an alternative question simulates that environment. Unlike in Experiment A, however, we do not get the censored responses from individuals who chose not to respond to the renaming-buildings question. These data are sensitive to the attractiveness of the other question. If the college-loans question is something that many or few of the sample would reply to, this will affect the effect size. The results are also sensitive to the college-loans question being *differentially* attractive to special groups, which has the potential to bias the sample in a way unrelated to our hypotheses.

Part 2 was a survey which asked primarily multiple-choice demographic questions. These included gender, age, race/ethnicity, preferred political party, and highest level of education. Part 2 was the same for both experiments.

### Coding for stance and frames

The survey gave text responses for the renaming-buildings question from participants in Experiment A and from those who chose this question in Experiment B. We manually coded text responses to the renaming-buildings question for both *stance* and for the *frames* used in the response. Based on its written content, every text response was assigned to one of five stance categories: pro (supported renaming buildings), con (opposed renaming buildings), neutral, conditional (it depends on the person/situation), and unclear (when we could not discern support). For the purposes of analysis, we focused mainly on the pro and con categories.

To create the framing codebook, each member of the research team initially independently coded 100 responses according to labels from Moral Foundations Theory [56], the Media Frames codebook [57], and with frames generated by the responses themselves. We then collectively tried to synthesize our frames into a set of consistent, reasonable codes. Ultimately, neither Moral Foundations nor the Media Frames Codebook aligned with our sample’s responses on renaming college buildings. So we developed and used our own set of codes through an iterative process: We coded a new set of responses using the previously created labels and with frames found in the new data. We then met and synthesized the codebook. This process repeated until the set of codes stabilized. Our codebook was informed by the other two sets of frames, but definitions are different. For instance, our definition of *harm* does not exactly match the one used in Moral Foundations.

Once the codebook was created, each author independently coded every response in sets of about 100 responses. After each set, we met to discuss our codes until a consensus was reached on every response. Coders were blinded, so we did not know the treatment condition or respondents’ demographics. Many responses had multiple frame codes. In the rare cases where there were more than three frames used in a response, we chose the three frame codes that were repeated the most often. In the case of ties, we chose the frames that were used earlier in the response. To calculate test-retest reliability, we performed this process again on a randomly chosen subset of 100 responses. This led to a test-retest reliability, using fuzzy kappa [58], of *κ* = .817.

**Frames.** Here is the list of frame codes and the criteria used:

**Erasing history**–Any reference to erasing history or rewriting the past.

**History as lesson–**Mentions how we can learn from history and/or historical building names.

**College’s role**–Refers to the college’s image, relationship between the college and the community, or the values of the college. Must explicitly mention the college.

**Cost**–Mentions a scarcity of resources, or the amount of work required to take an action.

**Progress**–Reference to moving on from a problematic past, making progress on social issues, or solving problems today that we had in the past. Includes metaphors of motion or growth from a past state.

**History is past**–History is in the past, and is therefore not important or less important than contemporary issues.

**Fairness**–Equal treatment or preferential treatment. Interpreted narrowly. For example, a reference to equality doesn’t automatically fall into this category.

**Same people, different times**–People are the same as they always have been. Or different times have different standards.

**Individuals’ contributions**–The specific contributions of the individuals who the buildings were named after should be considered. Includes references to relative contributions of different people, looking up to them as role-models, not honoring people who have done bad things, and references to worthiness due to monetary contributions.

**Unintended consequences**–There will be an unintended or surprising effect if buildings are renamed (or not renamed).

**Inconsistency**–There are inconsistencies in the present/future that would be created by renaming/not renaming. Typically referred to hypocrisy arising from some things being renamed when others aren’t.

**Different action**–Suggests a different action, besides renaming buildings.

**Harm**–Someone will be harmed in the present or future. Includes people taking offense, disrespect, damage to social well-being, and supporting students. Both increasing harm and reducing harm fall in this category.

**Authority**–Any reference to the individuals who have the right to make the decision.

**Doesn’t matter**–The decision to rename buildings will not have a practical impact. Or the discussion about renaming doesn’t matter.

Ad hominem*–Attacks the parties involved in the debate, rather than focusing on the merits of renaming. Includes criticizing their character, calling names, suggesting they are hypocrites, or implying they have the wrong mentality.

Challenges question*–Attacks the language used in the question or challenges the question itself.

Other*–Response unrelated to the question, using a frame not listed above, or no clear frame. Includes simple answers like “yes”. Originally coded as three categories: *off topic*, *other frame*, and *no frame*. However, it was hard to separate these categories, since these responses were often not clearly written.

*Any response that included either the *ad hominem*, *challenges question*, or *other* frame was coded as a *low-quality* response.

To test for differences in proportions, we used Boschloo’s test [59] using the Exact library [60] in R [61]. The Fisher exact test is inappropriate to analyze contingency tables if column sums are not fixed by design. Boschloo’s test adapts Fisher’s approach by comparing p-values across different column sums. It is uniformly more powerful than Fisher’s design. All Boschloo’s tests were one-tailed. The Plotrix library [62] was also used for visualization.

### Comparing frames

We were interested in inferring whether two groups C and D, such as whites and non-whites, were likely to use a different set of frames in their responses. This statistical analysis is challenging, since each response may have used 0, 1, 2, or 3 frames. In addition, there is no obvious statistical model which might explain how the groups use different frames.

So we used a random assignment Monte Carlo approach to infer whether two groups had similar frame use. We assumed as a null hypothesis that membership in Group C and Group D was independent of the probability of using each frame. We created a sampling distribution under the null by first tossing out the original group labels. We then randomly assigned every response to either Group C or Group D, ensuring that simulated groups had the same size as the actual groups. We calculated the test statistic under this simulated division. This process was repeated until we had 10,000 simulated test statistics. Our p-value is the percentage of these simulated test statistics which are larger than the test statistic for the actual sample.

For a test statistic, we used a variant of the Kullback-Liebler (KL) divergence [63]. Let $p_{f}^{C}$ be the observed proportion of responses from Group C that use frame *f*. Set $p_{f}^{D}$ in a similar fashion. For the null hypothesis, let *q*_*f*_ be the proportion of responses in the complete sample *C*∪*D* that used frame *f*. Then, the test statistic is:

$$\sum_{f}p_{f}^{C}\;log\;(\frac{p_{f}^{C}}{q_{f}})+\sum_{f}p_{f}^{D}\;log\;(\frac{p_{f}^{D}}{q_{f}})$$

Note that this is not a true KL divergence, which is typically defined on a probability space where probabilities sum to one. In our case, each response can have multiple frames, so ∑_*f*_*q*_*f*_>1. However, like KL divergence, this test statistic does measure how different the observed group probabilities $p_{f}^{C},\;p_{f}^{D}$ are from the reference distribution *q*_*f*_ corresponding to the null hypothesis.

All respondents gave informed consent through a digital interface. The University of Michigan institutional review board approved this study.

## Experiment A results

Experiment A was designed to understand both the responses of people who would respond in an online forum, as well as responses from people who would avoid posting online. So we asked everyone in the sample to respond to the prompt, and then self-rate how likely they would be to respond to it in an online community.

For the purposes of this analysis, we defined someone as a *likely responder* if they said they would be *somewhat likely* or *very likely* to respond to the question. We used this group to understand what might actually be posted online.

Table 2 gives some results from Experiment A.

**Table 2:** Experiment A—likelihood of responding, stance, and response quality by treatment group and race.

	Whites			Non-Whites			Likely Responders		
	Racial Inequality	White Privilege		Racial Inequality	White Privilege		Racial Inequality	White Privilege	
Count	189	161		59	66		133	97	
Average self-reported likelihood of responding	0.169 (.11)	-0.255 (.11)	**	0.203 (.19)	0.288 (.18)				
% Supported renaming	48	24	***	42	42		64	38	***
% Opposed renaming	29	41	**	27	30		18	38	***
Low quality response	22	37	**	24	36	+	20	36	**

+ p < .1, * p < .05, ** p < .01, *** p < .001

Respondents rated their likelihood of responding on a scale from 2 = *very likely to respond* to -2 = *very unlikely to respond*. Values in parentheses are standard errors. P-values represent differences between treatment groups. Three individuals did not provide a race.

### Avoidance

Based on their self-reported likelihood of responding, whites were less likely to respond to the *white privilege* question than the *racial inequality* question (t(344) = 2.73, p = .003). In contrast, non-whites were not significantly more likely to respond to the *white privilege question* (t(121) = -0.33, p = .372).

### Stance

Because we had coded multiple categories for stance, we separately report the percentages of people who supported (pro) and opposed (con) renaming buildings. The other stance categories did not have enough responders to draw reliable conclusions.

Whites in Experiment A were less likely to support (p < .001) and more likely to oppose (p = .008) renaming buildings when the question was phrased in terms of *white privilege*. This overall shift in stance among whites was surprising. When asked about *racial inequality*, whites were 67% more likely to be supportive than opposing. However, when *white privilege* was mentioned, 74% more whites opposed renaming college buildings than supported it.

As with avoidance, the choice of *racial inequality* versus *white privilege* did not affect average support (p = .505) or opposition (p = 0.667) among non- whites. This reinforces previous work that shows individuals have different responses when primed to think about their own group compared with another group.

Among likely responders, the term *white privilege* significantly decreased support for renaming buildings. In the *white privilege* condition, support dropped by 26 percentage points (p < .001), and opposition increased by 20 percentage points (p < .001). Unlike the results for whites and non- whites, these differences are caused by differences in *who* would respond in addition to stance changes.

### Response quality

Framing the question in terms of *white privilege* increased the percentage of low-quality responses. This was true among whites (p = .001), non-whites (p = .069), and likely responders (p = .003). The percentages for all groups were similar, so the decreased significance among non-whites is likely due to a smaller sample size.

### Frames

**Fig 1:**
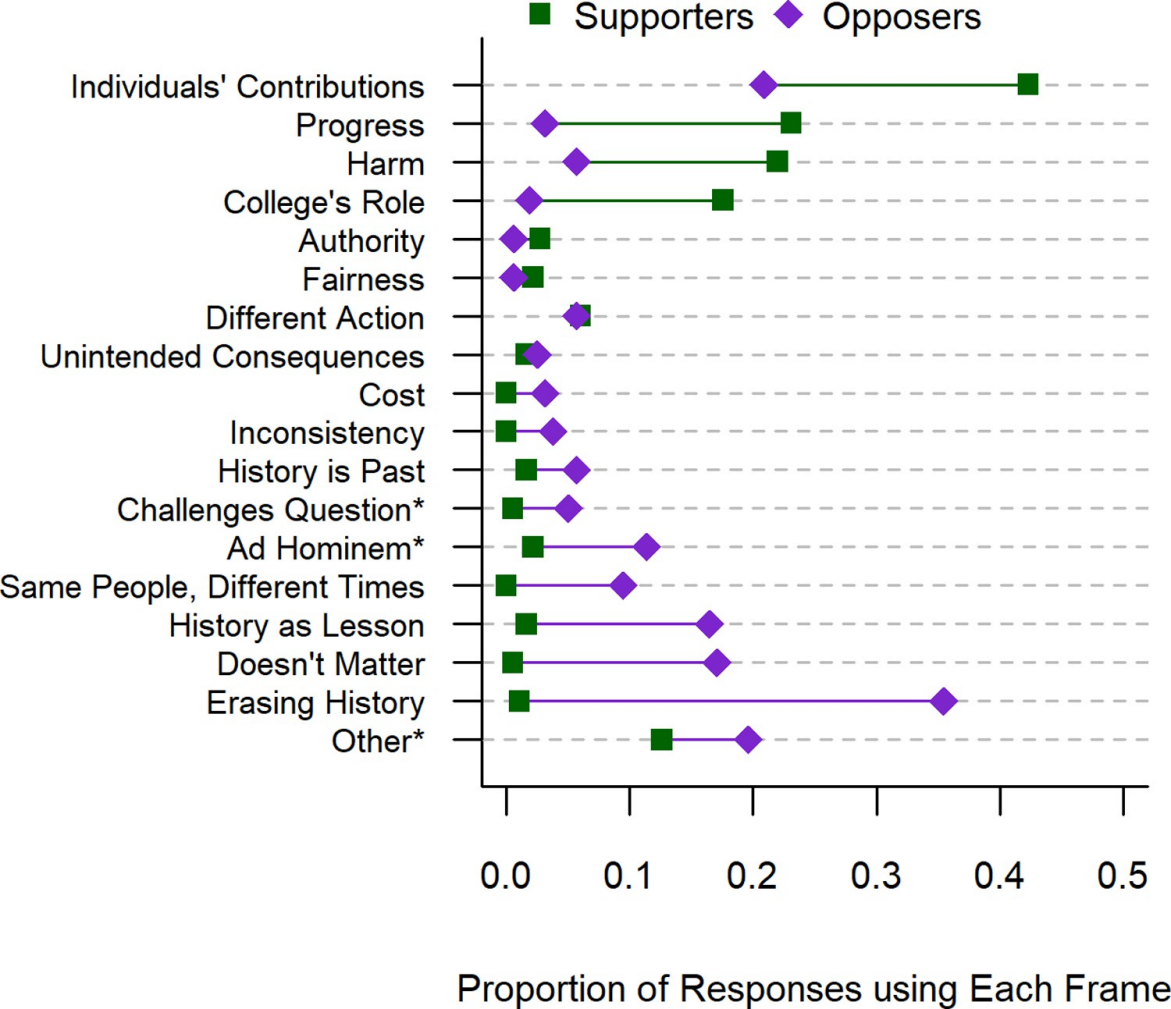
Percentage of responses in Experiment A that used each frame. Squares give the proportion of responses that used a given frame, among all responses that supported renaming buildings. Diamonds represent frame use among all responses that opposed renaming buildings. Starred frames were categorized as low-quality.

As predicted, the biggest difference in frame use was between supporters and opposers of renaming buildings (p < .001). The frequency of frame use for supporters and opposers is shown in Fig 1. We did not find a difference between the frames that whites and non-whites used in their responses (p = 0.768). This result held when we restricted the analysis to only those who received the *racial inequality* (p = 0.912) and *white privilege* (p = 0.649) questions.

Treatment condition did affect the frames that people used in their responses in both the complete sample (p = 0.018) and among likely responders (p = 0.029). Was this because the terms *racial inequality* and *white privilege* bring up different ideas in peoples’ minds? Or was it due to the fact that there are more supporters in the *racial inequality* condition, and supporting arguments generally use different frames?

To answer this, we performed a mediation analysis. We ran a logistic regression predicting the use of each frame based on treatment condition, controlling for support and opposition:

$$logit(F_{i})=\alpha +\beta (treatment_{i})+\gamma (pro_{i})+\delta (con_{i})+\epsilon_{i}$$

Here *F* indicates whether individual *i* used the chosen frame, *treatment* tells whether the individual received the *racial inequality* or *white privilege* question, and *pro/con* are binary variables that describe whether the individual supported or opposed renaming buildings. We ran this regression on every frame except the low-quality frames, which as described above did seem to show a difference between treatment conditions, and the *consistency* frame, which was used so rarely that the regression was not valid.

If the frames that people use in each treatment condition can be explained by their stance, then we would expect the coefficient of *treatment* to be uniformly distributed and mostly statistically insignificant. Though we do expect statistical significance (α = .05) to occur by random chance around 5% of the time. This is what we found. Of the 17 regressions only one frame, *erasing history*, had a p-value less than .05 (p = .014). The p-values seemed uniformly distributed, with the largest p-value for *authority* (p = .862). The effect of the term *white privilege* on framing was explained by individuals’ stances.

### Composition of responses

**Fig 2:**
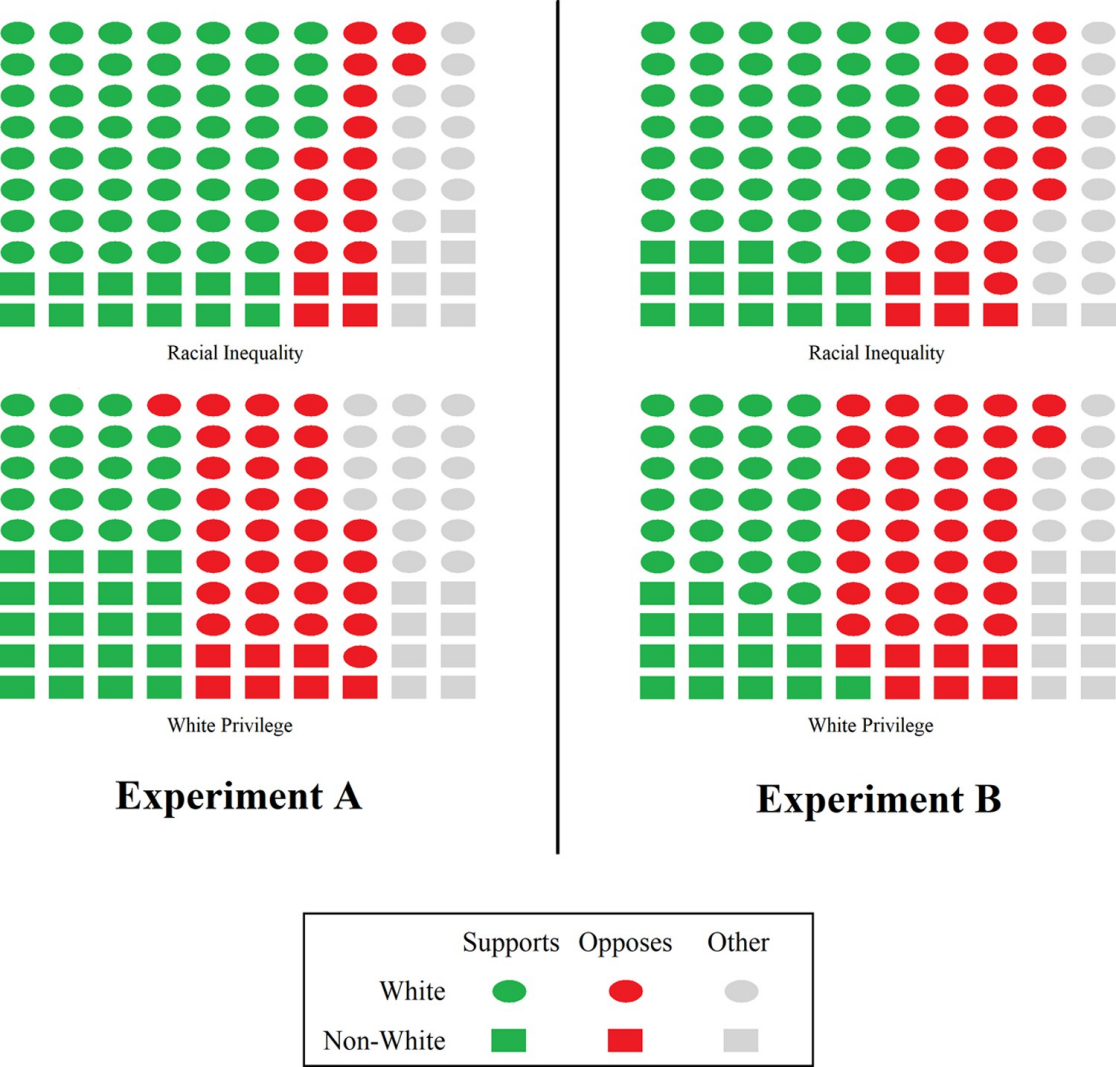
Composition of posts in a hypothetical online conversation among 100 responders who are representative of our sample. For Experiment A, the figure represents likely responders. For Experiment B, the figure represents those who responded to the renaming-buildings question. Shape corresponds to the race of each responder. Points are colored based on support for renaming buildings. The *Other* category includes responses that were neutral, unclear, or said that it should depend on the situation.

How does the question language affect the overall composition of responses that get posted online? We turn to the set of likely responders to analyze this question. Fig 2 gives a snapshot of what an online conversation might look like in each condition. The *racial inequality* question led to a set of likely responses that was overwhelmingly supportive of renaming buildings, with 7 supporters for every 2 opponents. In contrast, the *white privilege* framing led to a more divided set of responses, with roughly equal numbers of supporters and opponents. Different frames were brought up in the two conditions as well. Though, as mentioned, this seemed completely driven by differences in support. The *white privilege* question brought 80% more low-quality responses than the *racial inequality* question.

### Avoidance differences between whites

**Fig 3:**
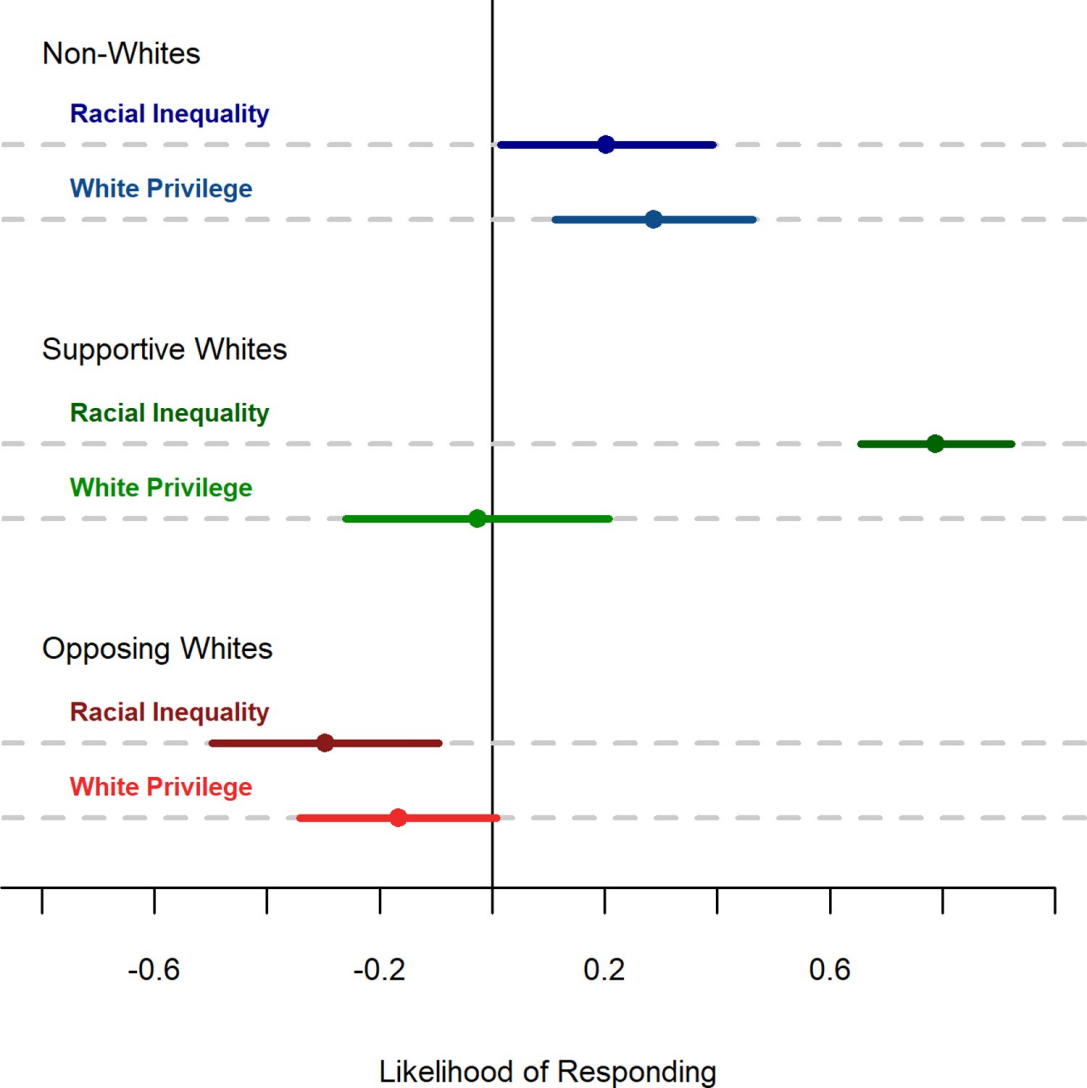
Average self-reported likelihood of responding in Experiment. **A.** Respondents rated their likelihood of responding on a scale from 2 = *very likely to respond* to -2 = *very unlikely to respond*. Error bars represent standard errors.

The effect of using the term *white privilege* did not affect all whites equally, as shown in Fig 3. Supportive whites were less likely to respond to the *white privilege* question than the *racial inequality* question (t(62) = 3.03, p = .004). However, whites who opposed renaming buildings were approximately equally likely to respond in both conditions (t(114) = -0.48, p = .635). Language choice did not affect the likelihood of responding among either supportive or opposing non-whites.

Overall, the results show that the shift from a set of overwhelmingly supportive responses under *racial inequality* to the divided responses under *white privilege* comes from two factors: (a) whites were, on average, less supportive of the *white privilege* question, and (b) supportive whites were less likely to respond to the *white privilege* question.

## Experiment B results

As a counterpoint to Experiment A, where people self-rated their likelihood of responding, Experiment B was designed to examine revealed behavior and see how people might respond in a simulated online environment. Respondents were given (a) the renaming-buildings question that corresponded to their randomly assigned treatment group and (b) the college-loans question. They were told to respond to only one of the questions.

37 respondents filled in the text boxes under both questions. This meant they provided a response for the college loans question, but that it was unclear whether they preferred to answer that question. Since our analysis focused on people who chose to respond to the renaming-buildings question over the college loans question, we excluded those 37 data points from the analysis in this section. For completeness, we performed a robustness check with those individuals included. The results were qualitatively similar to the results below but with smaller effect sizes.

The results in Table 3 tell a story consistent with the results from Experiment A. However, these results have generally weaker statistical significance. In particular, some of the effect sizes for non-whites seem to be similar to whites’ effect sizes, but without sufficiently small p-values. This is likely due to a smaller sample size. The alternate question about college loans seems to have been too attractive, with only about 1/3 of respondents answering the renaming-buildings question. This preference for the financial question over the race-related question held regardless of race or treatment condition, and warrants investigation in future studies.

**Table 3:** Experiment B—probability of responding, stance, and response quality by treatment group and race.

		Whites			Non-Whites			All Combined		
		Racial Inequality	White Privilege		Racial Inequality	White Privilege		Racial Inequality	White Privilege	
Count		163	152		49	44		213	196	
% Responding to Renaming Buildings Question		37	28	*	33	43		36	31	
Among those…										
	% Supported Renaming	54	38	+	62	47		56	41	*
	% Opposed Renaming	31	50	*	25	21		30	41	+
	% Low Quality Response	32	40	*	0	25	**	19	38	**

+ p < .1

* p < .05

** p < .01

*** p < .001

P-values represent differences between treatment groups. One individual did not provide a race.

### Avoidance

As in Experiment A, whites were less likely to respond to the *white privilege* question by nine percentage points (p = .035). Non-whites in the sample were 10 percentage points more likely to respond to the *white privilege* question (p = .160), but this did not rise to the level of statistical significance. So the effect for non-whites could be due to sampling variation. These results support Hypothesis 1.

### Stance

Whites who responded to the *racial inequality* question were, on average, more positive about renaming college buildings than those who responded to the *white privilege question*. They were 16 percentage points more likely to be supportive (p = .058) and 19 percentage points more likely to oppose (p = .030). Interestingly, non-white responders also seemed more positive about the *racial inequality* question. Though the sample size was small enough that neither the difference in support (p = .202) nor opposition (p = .427) were significant. When we consider the set of people who responded to the renaming buildings as a whole, the people who received the *racial inequality* question were more likely to be supportive (p = .043) and less likely to oppose (p = .091).

### Response quality

Responses to the *white privilege* question garnered a higher percentage of low-quality responses among whites (p = .047), non-whites (p = .010), and all responders (p =. 010).

### Frames

As in Experiment A, there was a large difference in frame use between supporters and opponents of renaming buildings (p < .001). There also was a significant difference in the frames between treatment conditions (p < .001). To analyze the effect of stance on frame use, we ran a logistic regression for each frame as described in Experiment A. The frames *unintended consequences* and *cost* were omitted from this analysis due to low use. The low-quality frames were also omitted. After controlling for stance, there was no effect of treatment condition on frame use beyond what we would expect by chance. The p-values were distributed fairly uniformly with the smallest p-value corresponding to the *consistency* frame (p = .040) and the largest corresponding to *erasing history* (p = .076). Again, the effect of question (*racial inequality/white privilege)* on frame use was completely explained by stance. These results support Hypothesis 4.

### Composition of responses

Fig 2 shows the overall composition of responses. As before, *racial inequality* led to more supportive responses and fewer low-quality responses than when the question was framed in terms of *white privilege*. As in Experiment A, there were equal numbers of supporters and opponents when asked about *white privilege*, and responders were generally supportive when asked about *racial inequality*. There were 1.9 supporters for every opposer in the *racial inequality* condition. This was weaker than in Experiment A, where the support/opposition ratio was 3.5. It is unclear whether this weaker support is caused by the attractiveness of the college-loans question, a difference between stated preferences (Experiment A) and revealed preferences (Experiment B), or random chance.

### Summary of results

These results shed light on our hypotheses. Hypothesis 1 and Hypothesis 2 are both confirmed by the data. Whites who received the *white privilege* questions were less likely to respond and less supportive of renaming buildings. We also found support for Hypothesis 3. Use of the term *white privilege* led to more low-quality responses. This result was not only true among whites, but also among non-whites. The results also support Hypothesis 4, which focused on motivated reasoning. Supporters and opponents of renaming college buildings used different arguments. However, differences in framing between people who received the *white privilege* and *racial inequality* question disappeared after taking into account their stance. These experiments also provided evidence for Hypothesis 5. The term *racial inequality* created a set of responses that supported renaming college buildings. *White privilege* led to a more divided, polarized set of posts. While the effects of the term *white privilege* on whites was unambiguous, the effect on non-whites was less clear due to a combination of smaller sample sizes and seemingly weaker effects. The only reliable result among non-whites was that *white privilege* led to more low-quality responses.

## Discussion

Using two experiments, we studied how individuals respond to the term *white privilege* in an online environment. Mentioning *white privilege* was enough to flip white support for renaming college buildings from primarily supportive to primarily opposing. Furthermore, the term *white privilege* deters some supportive whites from engaging in the conversation. Surprisingly, we did not see this avoidance effect among opposing whites. In addition, the term *white privilege* led to less constructive responses among both whites and non-whites.

If these were posts on a real online discussion board, asking about *racial inequality* would give the impression of general support for renaming college buildings. Asking about *white privilege* would lead to a seemingly less supportive, more divided public opinion with lower-quality online debate. This decreased support is driven by two factors: (a) whites were, on average, less supportive when *white privilege* was brought up, and (b) supportive whites were more likely to avoid talking about *white privilege*.

Responses to *white privilege* tended to use different arguments from arguments about *racial inequality*. However, that difference was completely explained by differences in stance toward renaming buildings. This lends credence to the claim that the term *white privilege* leads first to a change in stance, followed by motivated reasoning to support that stance. If the causality went the other way, where the choice of language first affects the ideas people have, which leads to them changing their support, then we might expect at least some of the frames to be unexplained by stance.

Prior literature suggests that both emotion [28, 64] and the strength of racial identity [18] play a significant role in our results. We hypothesize that the increased tendency of supportive whites to avoid discussing *white privilege* is mediated by both these factors. It could be that the term made racial identity more salient for all whites, but was more likely to generate guilt and therefore avoidance in supportive whites. Another possibility is that opposing whites tended to identify highly with their race already, so that mentions of *white privilege* had a greater average effect on both racial identity salience and emotion on lower-identifying whites. Future research might test these hypotheses.

In writing about this study, we had to refer to groups, such as “non-whites” and “supportive whites”. There is a lot of variation among the individuals in any group, especially racially-defined groups with millions of members. However, humans have an unfortunate tendency to generalize a statement about a group of people to each individual member [25]. This overgeneralization can cause harm, for instance through stereotyping [65]. Our study, like many research studies, is about averages. So we have been careful to use language that minimizes overgeneralization to individuals. For instance, instead of writing, “Whites were less supportive of the *white privilege* question”, we wrote “Whites were, *on average*, less supportive of the *white privilege* question.” Our results should be interpreted as describing how language affects large-scale social dynamics, not as a way to understand traits or behaviors of individuals.

### Limitations

In a real online site, social desirability bias, the design of the forum, and back-and-forth between posters may magnify or dampen the effects we saw here. Another limitation comes from the fact that most social media users post very rarely. Online, the desires for information and entertainment are major drivers of behavior. Indeed, some researchers emphasize the value of active listening [66], which can bring a more diverse set of perspectives. All participants in our study were motivated to respond. It is unclear how the desire to read others’ points of view might affect these results. In addition, Experiment A and Experiment B had quantitatively different but qualitatively similar results. So in a true online environment, we might expect a similar effect, but with potentially different effect sizes.

The present study does not capture long-term attitude changes. Further research is required to understand the circumstances under which long-term exposure to the term *white privilege* affects support for racially progressive policies, whether it increases animosity and polarization, and how this effect might differ between demographic groups.

While we chose the language in the study to broadly evoke group-based identity, the terms *racial inequality* and *white privilege* do have different literal meanings. The survey prompt asked individuals to think about buildings named after people who supported these two separate concepts. It’s not clear whether that difference in meaning affected their responses. Concerns about building names have cited a variety reasons, from the honoree being a Confederate to supporting eugenics. Perhaps *white privilege* and *racial inequality* suggest different reasons, which led to different responses by treatment group.

### Implications

Our study has several practical implications. The first is already known, but often ignored: Opinions on social media do not represent public opinion. Social media posts are highly dependent on how a question is phrased, as well as the norms, community members, and moderation practices of the site. Individual and system-level forces, such as self-categorization [25], the spiral of silence [54], and algorithmic filters [67] affect what shows up on our feeds. In our study, which did not include the moderation found on social media platforms, a two-word change in language was sufficient to shift a community from appearing divided to appearing supportive. This result will not be surprising to survey researchers, who need to be very attentive to choice of language [68]. However, policy-makers [52], journalists [51], and others who use social media to understand the opinions of others may want to turn to more valid sources.

Those who want inclusive online conversations around race and/or support for racially sensitive policies should think carefully about the use of language like *white privilege* that targets the racial identity of specific groups. This language can deter the targeted group from participating. It has the potential to increase affective polarization by creating the image of a politically divided online space. Using slightly different language, such as *racial inequality*, that has more of a shared meaning across cultures can lead to conversations with broader participation and greater shared support.

In discussing this study with academic colleagues, a common response was, “Even if the term *white privilege* makes whites feel uncomfortable, they still need to hear it. It’s part of learning about race.” Indeed, numerous scholars have argued for raising awareness of race-based privilege [9]. Spending time thinking about racial advantages and disadvantages can affect individuals’ perceptions of systemic discrimination [23, 69]. However, these effects vary significantly depending on the details of the intervention and the individuals involved [9, 23, 69]. Our results, which focused on a simple change of language in an impersonal context, show that mention of *white privilege* can decrease engagement and lead to opinion shifts opposite to what was intended. It’s reasonable to expect that this identity-based disengagement decreases learning for some whites–an effect which has been documented in other settings [70–72]. Humanity has an evolutionarily useful, but usually incorrect, tendency to treat all members of a group as being the same [25, 73]. As commonly used, the phrase *white privilege* draws on this tendency to conflate individual traits with group averages, in a way that creates unpleasant emotions. A more effective approach might be to distinguish between individuals’ experiences and group averages through a combination of personal storytelling and large-scale data in a way that is consciously inclusive of whites [74].

## Conclusion

With online political polarization on the rise [75] and race in the forefront of today’s news, it is important to make cross-cultural online communication effective and inclusive. The present work adds to what we know about communication on racially challenging topics. This study has shown that the term *white privilege* in online conversations tends to decrease support for racially ameliorative policies among whites, cause some supportive whites to avoid participating in discussions, decrease overall online conversation quality, and lead online forums to seem more polarized. Other, more inclusive, ways of speaking about race online, such as the term *racial inequality* are more likely to create a sense of shared purpose. There are very real racial inequities in society today. Choosing language that promotes constructive conversation will not solve those problems. But it is an important step towards collectively understanding their dimensions and working together towards a solution.

## Supporting information

S1 File R code for replication of results. (R)

S1 Data Data for use with the replication code. To ensure participants’ privacy, data for *Table 1: Demographics of respondents* has been omitted.
(CSV)

S2 Data Names and descriptions of the variables found in the data. (TXT)
